# Effect of nitrogen levels and nitrogen ratios on lodging resistance and yield potential of winter wheat (*Triticum aestivum* L.)

**DOI:** 10.1371/journal.pone.0187543

**Published:** 2017-11-08

**Authors:** Mingwei Zhang, Hui Wang, Yuan Yi, Jinfeng Ding, Min Zhu, Chunyan Li, Wenshan Guo, Chaonian Feng, Xinkai Zhu

**Affiliations:** 1 Laboratory of Crop Genetics and Physiology of Jiangsu Province / Co-Innovation Center for Modern Production Technology of Grain Crops / Wheat Research Institute, Yangzhou University, Yangzhou, China; 2 Lixiahe Insitute of Agricultural Sciences of Jiangsu, Yangzhou, Jiangsu, China; Institute of Genetics and Developmental Biology Chinese Academy of Sciences, CHINA

## Abstract

Lodging is one of the constraints that limit wheat yields and quality due to the unexpected bending or breaking stems on wheat (*Triticum aestivum* L.) production worldwide. In addition to choosing lodging resistance varieties, husbandry practices also have a significant effect on lodging. Nitrogen management is one of the most common and efficient methods. A field experiment with Yangmai 20 as research material (a widely-used variety) was conducted to study the effects of different nitrogen levels and ratios on culm morphological, anatomical characters and chemical components and to explore the nitrogen application techniques for lodging tolerance and high yield. Results showed that some index of basal internodes, such as stem wall thickness, filling degree, lignin content, cellulose content, water-soluble carbohydrate (WSC) and WSC/N ratio, were positively and significantly correlated with culm lodging-resistant index (CLRI). As the increase of nitrogen level and basal nitrogen ratio, the basal internodes became slender and fragile with the thick stem wall, while filling degree, chemical components and the strength of the stem decreased gradually, which significantly increased the lodging risk. The response of grain yield to nitrogen doses was quadratic and grain yield reached the highest at the nitrogen ratio of 50%:10%:20%:20% (the ratio of nitrogen amount applied before sowing, at tillering stage, jointing stage and booting stage respectively, abbreviated as 5:1:2:2). These results suggested that for Yangmai 20, the planting density of 180×10^4^ha^-1^, nitrogen level of 225 kg ha^-1^, and the ratio of 5: 1: 2: 2 effectively increased lodging resistance and grain yield. This combination of planting density and nitrogen level and ratio could effectively relieve the contradiction between high-yielding and anti-lodging.

## Introduction

Wheat is one of the most important agricultural crops in the world. Achieving high wheat yields in both irrigated and rain-fed environments has been limited by the disorders known as lodging [1, 2], defined as the permanent displacement of plant shoots from an upright position [3]. Lodging is one of key constraints limiting wheat yields and quality in both developed and developing countries [4, 5], due to reducing photosynthetic capacity, providing a favorable environment for fungal growth and leaf disease development [6].

Lodging occurs due to the interactions between plant, wind, rain and soil. Wind and rain exert a force which bends or breaks the stem base (stem lodging), or displaces the roots within the soil (root lodging) [7]. There was a conjecture about which type predominates with the review of lodging by Pinthus [3] favoring root lodging and Neenan and Spencer-Smith [8] reasoning that stem failure to be the more common cause of lodging. But in China, stem lodging is more common. Lodging issue could reduce grain yield up to 20%- 40%, with the magnitude of loss dependent on the cultivar, growth stage and severity of lodging [3, 9]. Reductions in kernel number, kernel weight has been reported as a direct result of lodging stress in winter wheat [10, 11]. Severe lodging occurred in UK cereal crops as well, on average, every 3 or 4 years when 15–20% of the wheat area is waterlogged [1]. However, in China [12], plant growth regulators have not been widely used to improve lodging resistance in high yielding environments, thus lodging is the most important constraint limiting high yield [13]. According to the survey, yield losses caused by lodging is as high as 2 billion kg in China.

Plant breeders have reduced lodging risk by introducing dwarfing genes to decrease plant height which is negatively correlating with lodging resistance [14]. But the reduction in plant height to improve lodging resistance could reduce the photosynthetic capacity resulting reduction of yield. While the basal part of the culm internode also plays an important role in lodging resistance as it provides a lever to hold the plant upright. Improving the physical strength of the basal part in the culm internode is necessary completely [15]. Previous studies showed that lodging resistance was significantly correlated with culm morphological, anatomical characters and chemical components [16, 17]. Length of basal internodes were negatively correlated with lodging resistance, while culm diameter, wall thickness, and dry weight per cm of basal internodes were positively associated with lodging resistance [18]. Likewise, significant correlations were found between lodging resistance and several anatomical features, including cell layer of sclerenchyma (mechanical tissues), the number of vascular bundlers and the area of single vascular bundler, and pith diameter [19]. Several culm chemical components were positively related with lodging resistance as well. Kokubo et al. [20] reported that high correlations between the cellulose content of cell walls and the maximum bending stress, and found that silica deposited in the epidermis of wheat culms were more abundant in a lodging resistant variety than in a lodging susceptible variety. In addition, Chen et al. [21] advanced the view that the accumulation of lignin and hemicellulose of culms in lodging wheat varieties were lower than that in lodging-resistant varieties. Increases in lignin content could significantly improve the mechanical intensity of culm [22].

Plant breeders and farmers have to keep improving lodging resistance to counter the escalating lodging risk arising from continued yield increases. Plant breeders have reduced lodging risk by introducing dwarfing genes to produce shorter varieties [12]. It was not the only choice of cultivar that affects the incidence of lodging: crop management is also important [23]. The common strategy is to utilize plant growth regulators to shorten crops. In UK, plant growth regulators were applied to 88% of the winter wheat area in 2004 [12]. However, in China, growth inhibitors has not been commonly used. While other lodging-avoidance methods that include reducing seed rate, delaying sowing, rolling the soil and reducing/delaying nitrogen are widespread used in Chinese wheat production [12]. Among them, Nitrogen regulation is the most convenient and effective method that affects the stem traits and influences the lodging resistance in plants. Knapp et al. [24] have reported that augmenting nitrogen rates resulted in 10–25% increase in the length of basal internodes, which contributed to lodging. Ali [25] also found that utilization of more nitrogen decreased breaking strength of the second internode significantly. Berry et al. [7] have suggested reducing spring nitrogen could reduce the height of gravity and increased stem strength by increasing both stem diameter and wall width. Crook et al. [23]also found that strong-stemmed plants resulting from wide stem bases with thick walls may be produced by applying less nitrogen. Wei et al. [26] have reported that the cell wall cellulose and lignin contents increased at first and then descended with the increase of nitrogen levels. Grain yield response to nitrogen doses was quadratic, and linear increase in lodging. So splitting nitrogen fertilization increased lodging and brought no benefit to grain yield. Several studies have reported that lodging reductions have been achieved with sacrificing yield potential by decreasing nitrogen level and delaying nitrogen fertilizer applications [7, 27–28]. But it remained unclear whether nitrogen management could reduce lodging risk without reducing yield potential.

Yangmai 20, a high-yielding winter wheat variety, is widely planted in the middle and lower reaches of the Yangtze River. This variety is a medium-gluten type which is used for dumplings and noodles. Yangmai 20 has high yield potential, while the performance of lodging resistance is unstable in different years, which is the key factor influencing wheat yield.

The influence of nitrogen rates to the lodging resistance of wheat have been reported by several studies. However, it is not clear how far the effects of nitrogen ratios could be additive, or the degree to which the combined effects of nitrogen rates and nitrogen ratios would make the plant be resistant to lodging. Additionlly, little information was about the optimal combination of nitrogen rate and ratio, which could improve lodging resistance without reducing yield potential. Accordingly, the trial was conducted to study the effects of different nitrogen levels and nitrogen application ratios on lodging resistance and investigate the relationship between plant morphology, stem anatomical structures, chemical components and other indicators with lodging resistance and to explore the nitrogen application techniques for anti-lodging and high-yielding.

## Materials and methods

### 2.1 Experimental location and growth conditions

**Fig 1:**
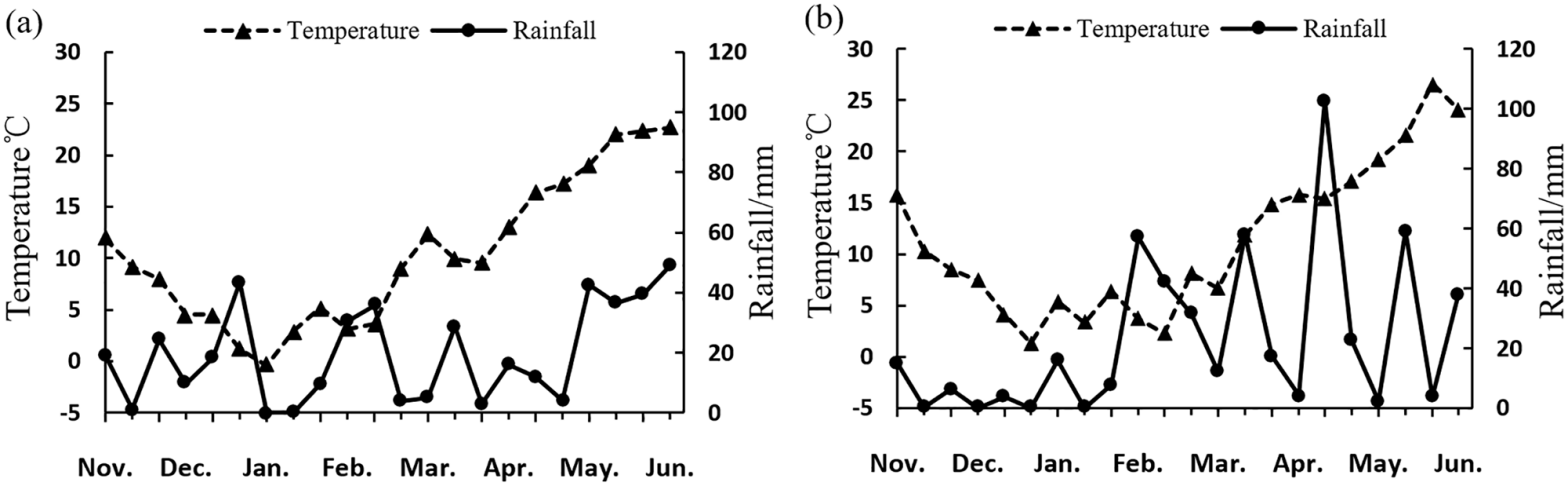
Daily temperature and rainfall regimes during growing season at the experimental site in 2012–2013 (a) and 2013–2014 (b).

A field trial was designed at the Agricultural Experiment Station (32°39’E, 119°42’N) of Agricultural College of Yangzhou University in China during the growing seasons of 2012–2013 (2012) and 2013–2014 (2013). The experiments were performed in the field with winter wheat–rice rotation, where the soil was a light loam with mean contents of 72.16 mg kg^–1^ available N, 52.20 mg kg^–1^ available P, and 157.24 mg kg^-1^ available K at 0 to 20 cm upper soil layer. The experiments were carried out in a zone of humid subtropical climate with an annual average temperature of 13.2°C–16.0°C, total precipitation of 800 mm–1200 mm, total sunshine of 2000 h–2600 h, and a frost-free season of 220 d–240 d. Weather conditions during the wheat growing season (Fig 1) were close to normal.

### 2.2 Plant materials and experimental design

A high-yielding winter wheat cultivar currently used in local production, Yangmai 20, was chosen in the experiment. This cultivar was obtained from the Agricultural Science Institute of the Lixiahe District. The experiments were laid out in a split-plot design with three replicates. Main plots were total nitrogen (N) levels, including 180, 225, and 270 kg ha^-1^. Subplots were nitrogen ratios, including 70%:10%:20%:0% (labeled as 7:1:2:0), 50%:10%:20%:20% (labeled as 5:1:2:2) and 30%:10%:30%:30% (labeled as 3:1:3:3) applied at four stages. The 1st, 2nd, 3rd and 4th nitrogen application was applied pre-sowing, the four-leaf stage (DC 14) [29], at the jointing stage (DC 32), and booting stage (DC 45) respectively. The total doses of P_2_O_5_ (P) and K_2_O (K) were both 120 kg ha^-1^. Half the amounts of P_2_O_5_ (P) and K_2_O (K) were applied before sowing, and the remaining half was applied at the jointing stage (DC 32). Before sowing, the basic fertilizer was incorporated into the soil by cultivation, and other fertilizers were used as top dressing. Inorganic compound fertilizers (containing 15% N, 15% P_2_O_5_ and 15% K_2_O) and urea (containing 46.7% N) were preferentially applied, and triple superphosphate (containing 12% P_2_O_5_) and potassium chloride (containing 50% K_2_O) were added to meet the fertilizer requirement. No organic manure was applied. The plot dimensions were 3 m × 3 m with 10 rows (0.3 m between rows). It sown at a planting density of 180×10^4^ ha^-1^ with a line on Nov. 1, 2012, and Oct. 28, 2013. The seedling number was controlled to 180×10^4^ ha^-1^ by removing or adding seedlings at the three-leaf stage (DC 13). Plants were harvested on June. 3, 2013 and June. 1, 2014. Other cultural practices followed the precise high-yielding cultivation system of Cheng [30].

### 2.3 Measurement items and methods

#### 2.3.1 Lodging score

Lodging was assessed by measuring the lodging rates and the angle of inclination of the stem base from the vertical with a score calculated following the method of Fischer and Stapper [4], namely, lodging score = lodging rates ×angle of lodging from vertical position / 90. Lodging rates (%) was scored with the formula ((the lodging area in plot/the plot area) × 100%) at maturity of three replications, as described by Chen et al. [21].

#### 2.3.2 Pushing resistance determination

Five culms from a single row in each plot were used to measure pushing resistance from the center of gravity at milk stage (DC 73) with a prostrate tester (FG-5005, SATO SHOUJI INC., Japan). Pushing resistance of the lower stem part at right angles to the row direction was measured when plants were forced to 45° relative to the ground [31].

#### 2.3.3 Culm breaking strength and culm lodging resistant index (CLRI)

Ten standing plants were selected randomly from each plot at milk stage (DC 73), avoiding the outer two rows, to measure the height at center of gravity (HCG) and culm breaking strength of the 2^nd^ internode (CBS). The CBS of basal second internodes was measured with a plant lodging tester (YYD-1A, Zhejiang TOP Instrument Co., Ltd, China). Put the sampled basal second internode with removed stem sheath on the groove of support pillars with a distance of 5 cm. The tester was set perpendicular to the stem at the middle, loading gradually, and CBS was measured when culm internode was pushed to break [15]. HCG was determined by cutting off the roots and balancing the isolated shoot on a ruler (leaves and ear still attached). The distance from the point of balance to the base of the stem was recorded [7]. We then calculated the culm lodging resistant index (CLRI) in milk stage (DC 73). The methods for these measurements are described by Li et al. [32]. The culm lodging resistance index was calculated as following: Culm lodging resistant index = equations: culm breaking strength of basal second internodes/ culm height at center of gravity.

#### 2.3.4 Plant height, diameter, wall thickness, filling degree

Twenty representative stalks per plot without stem damage were collected to measure the length, diameter, wall thickness, filling degree of the basal second internode and the plant height at DC 73 according to the methods in Wei et al. [26]. Plant height was measured from the plant base to the spike tip excluding awns. After detaching the leaves along with the leaf sheath, diameter and wall thickness of basal second internode (measuring on the mid of the internode) were measured with a digital caliper having accuracy of 0.001 mm. Filling degree was scored with the formula (the dry weight of basal second internode/the length of basal second internode) [31].

#### 2.3.5 Water-solute carbohydrates (WSC) and nitrogen determination

Twenty culms of similar height were selected and uprooted from the middle rows of each plot at DC73. For each sample, the lower internodes were placed in a fan-driven dehydrator (Memmert UF750) and dried at 80°C for 48 h. The dried samples were ground to pass through a 1 mm sieve in a pulverizer and then used to extract water-solute carbohydrates (WSC) using boiling deionized water. WSC concentrations were quantified by colorimetry using anthrone reagent [33]. Concentrations of N were digested H_2_SO_4_–H_2_O_2_ solution and determined by the Micro-Kjeldahl method.

#### 2.3.6 Lignin, cellulose and Silicon content determination

Plant samples were treated similarly in both two years. The twenty randomly culms were selected from each plot at milk stage (DC73). The basal internodes were oven dried at 80°C and then ground to pass through a 1 mm sieve. Tissues from the basal internodes from three replications were analyzed for cellulose content, lignin content [34] and silicon content [35].

#### 2.3.7 Yield and its component

At maturity (DC93), an area of 1.2 m × 1 m per plot of the center four rows was manually harvested for each experiment, avoiding edge rows on the each plot end. Then the grain weight was determined after 1–2 weeks of sun drying at the standard 130 g kg^-1^ moisture content, which is used for grain yield designation throughout. At the time of harvest, number of spikes was counted manually for three rows within 1 m. Fifty spikes from each plot was taken in succession and its number of kernels per spike was recorded. For 1000-kernels weight, 1000 kernels were randomly counted and weighed.

### 2.4 Statistical analyses

Analysis of variance (ANOVA) was performed using Statistical Product and Service Solution 15.0 for Windows (SPSS Inc., Chicago, IL, USA) for all traits. The significance of each source was determined by F-test. Least significant difference (LSD) tests were performed to determine significant differences between individual means. Simple correlation coefficients between the characters examined were calculated using correlation analysis. The relationships between culm traits and culm lodging resistance index (CLRI) were revealed by grey relational analysis described in the SPSS.

## Result

### 3.1 Apparent lodging rate, lodging score (LS) and yield of wheat

As shown Table 1, the lodging period and degree between two years is different due to the climate variation, but the laws are similar. Along with the increase of nitrogen level and the proportion of basal fertilizer, the lodging risk also increased significantly, and an escalating trend is manifested in both the lodging degree and area. The weather condition during 2012–2013 was normal, and the lodging was not serious, which occurred mainly after the grouting. Under nitrogen ratio of 7: 1: 2: 0 (the ratio of N applied before sowing, at tillering stage, jointing stage and booting stage), the lodging stress was quite severe. When the nitrogen application amount was 270 kg ha^-1^, the lodging angle of 80% area was above 70°. The weather condition during 2013–2014 was abnormal, heavy rains and winds were quite often after anthesis (DC 61) of wheat, so the lodging occurred inevitably. With the increase of nitrogen level and the proportion of basal fertilizer, the lodging degree, area and lodging score (LS) showed an increasing trend. The lodging with nitrogen ratio of 7:1:2:0 treatments under different nitrogen application amounts were the most serious. Under 270 kg·ha^-1^ application level of nitrogen fertilizer, the angle of different nitrogen ratios treatments was above 70°, with the area of more than 50%, and the mean value of LS to be 50.51.

**Table 1:** Effect of nitrogen levels and ratios on apparent lodging rate, lodging score and grain yield.

Year	N level (kg ha ^-1^)	N ratio	Lodging stage ^α^	Lodging angle (°)	Lodging rate (%)	Lodging score	Spikes (10^4^ per ha^-1^)	Kernels per spike	1000-kernels weight (g)	Grain yield (kg ha ^-1^)
2012–2013	180	7:1:2:0	NL	0	0	0	447.08d ^β^	44.02ab	38.88bc	7072.22d
		5:1:2:2	DC83	15	10	1.67	494.72abc	41.19bc	40.72a	7708.33abc
		3:1:3:3	NL	1	0	0	462.50cd	42.92abc	40.18ab	7391.67bcd
	225	7:1:2:0	DC83	70	40	32.22	506.67ab	44.58a	35.28de	7233.33cd
		5:1:2:2	DC87	20	17.5	3.89	533.33a	45.34a	36.03d	8075.00a
		3:1:3:3	NL	0	0	0	477.50bcd	45.73a	38.67c	7916.67ab
	270	7:1:2:0	DC75	72.5	80	71.11	509.72ab	40.34c	34.10e	6950.00d
		5:1:2:2	NL	0	0	0	507.50ab	43.03abc	35.99d	7775.00ab
		3:1:3:3	NL	0	0	0	497.22abc	40.78c	36.65d	7400.00bcd
2013–2014	180	7:1:2:0	DC65	50	32.5	18.06	452.24d	41.95b	37.75ab	7091.67cd
		5:1:2:2	DC65	45	15	7.5	478.36bc	42.68ab	37.58b	7650.00abc
		3:1:3:3	DC73	20	15	3.33	451.69d	43.86ab	39.01a	7633.33abc
	225	7:1:2:0	DC65	72.5	55	44.31	484.47bc	43.75ab	36.51b	7216.67bcd
		5:1:2:2	DC65	65	22.5	16.25	508.91a	43.40ab	36.81b	7833.33a
		3:1:3:3	DC65	40	12.5	5.56	500.03ab	44.52ab	37.05b	7933.33a
	270	7:1:2:0	DC65	85	65	57.78	469.20cd	43.57ab	36.92b	6991.67d
		5:1:2:2	DC65	77.5	55	44.31	474.19cd	44.73a	37.48b	7791.67ab
		3:1:3:3	DC65	70	55	41.25	464.47cd	44.66ab	37.73ab	7519.44abcd

^α^ NL: No lodging; DC65: Anthesis; DC73: Early milk stage; DC75: Medium milk stage; DC83: Early dough stage; DC87: Hard dough stage

^β^ Values in a column followed by the same letter are not significantly different at P < 0.05 as determined by the LSD test. The test was conducted separately between the two years.

In this experimental condition, nitrogen level and ratio had a significant effect on grain yield and its components of Yangmai 20 (Table 1). Grain yield first increased then decreased with the increase of the total amount of nitrogen fertilizer, and achieved the highest grain yield at 225 kg ha^-1^ nitrogen level, which respectively increased by 3.76% and 4.01% compared with the nitrogen levels of 180 kg ha^-1^ and 270 kg ha^-1^. It was suggested that the moderate increase of nitrogen level was beneficial to improve grain yield, but splitting nitrogen fertilizer would deteriorate the population structure, increase the risk of lodging and decrease the yield. The treatment of 5:1:2:2 (the ratio of nitrogen applied before sowing, tillering, jointing and booting stage) reached the highest grain yield, which increased by 10.05% and 2.27% compared with the treatments of 7:1:2:0 and 3:1:3:3, respectively.

### 3.2 Culm breaking strength and culm lodging resistant index (CLRI)

The culm breaking strength and culm lodging resistant index (CLRI) decreased with the increase of nitrogen application rate during 2012–2013, under the same nitrogen application rate, both of the two traits increased with the increase of topdressing fertilizer ratio (Table 2). Both the culm breaking strength and CLRI were lower significantly in the case of lodging than in non-lodging treatments, with the greater lodging angle and the larger area. The correlation analysis indicates that CLRI had a significant linear correlation with LS.

**Table 2:** Effect of nitrogen levels and ratios on culm breaking strength and culm lodging resistance index.

N level (kg ha ^-1^)	N ratio	2012–2013			2013–2014		
		Lodging score	Culm breaking strength(Newtons)	Culm lodging resistance index	Lodging score	Culm breaking strength(Newtons)	Culm lodging resistance index
180	7:1:2:0	0	13.57abc ^α^	25.81ab	18.06	13.84ab	26.00ab
	5:1:2:2	1.67	16.36a	32.02a	7.5	15.52a	29.43a
	3:1:3:3	0	16.74a	33.01a	3.33	15.49a	29.83a
225	7:1:2:0	32.22	12.10bc	23.24b	44.31	12.77ab	24.06ab
	5:1:2:2	3.89	15.32abc	29.72ab	16.25	14.39ab	27.12ab
	3:1:3:3	0	15.78ab	30.84ab	5.56	15.21a	28.91ab
270	7:1:2:0	71.11	11.81c	22.43b	57.78	11.19b	20.83b
	5:1:2:2	0	12.99abc	25.28ab	44.31	12.60ab	23.48ab
	3:1:3:3	0	13.15abc	25.63ab	41.25	13.16ab	24.40ab
F							
Nitrogen levels(NL)			9.85**	8.46**		7.34**	8.84**
Nitrogen ratio(NR)			10.16**	10.40**		0.99	4.99*
NL×NR			0.69	0.56		1.96	0.07

^α^ Values in a column followed by the same letter are not significantly different at P < 0.05 as determined by the LSD test. The test was conducted separately between the two years.

* F-test significant at 5% level.

** F-test significant at 1% level.

Data of the two-years showed that the nitrogen fertilizer had a significant effect on the culm breaking strength and CLRI. The increase of nitrogen level could lead to the decrease of the lodging resistance. Therefore, reducing the application amount of nitrogen fertilizer moderately, with postponing nitrogen application could relief the lodging stress.

### 3.3 Pushing resistance of the stem

**Fig 2:**
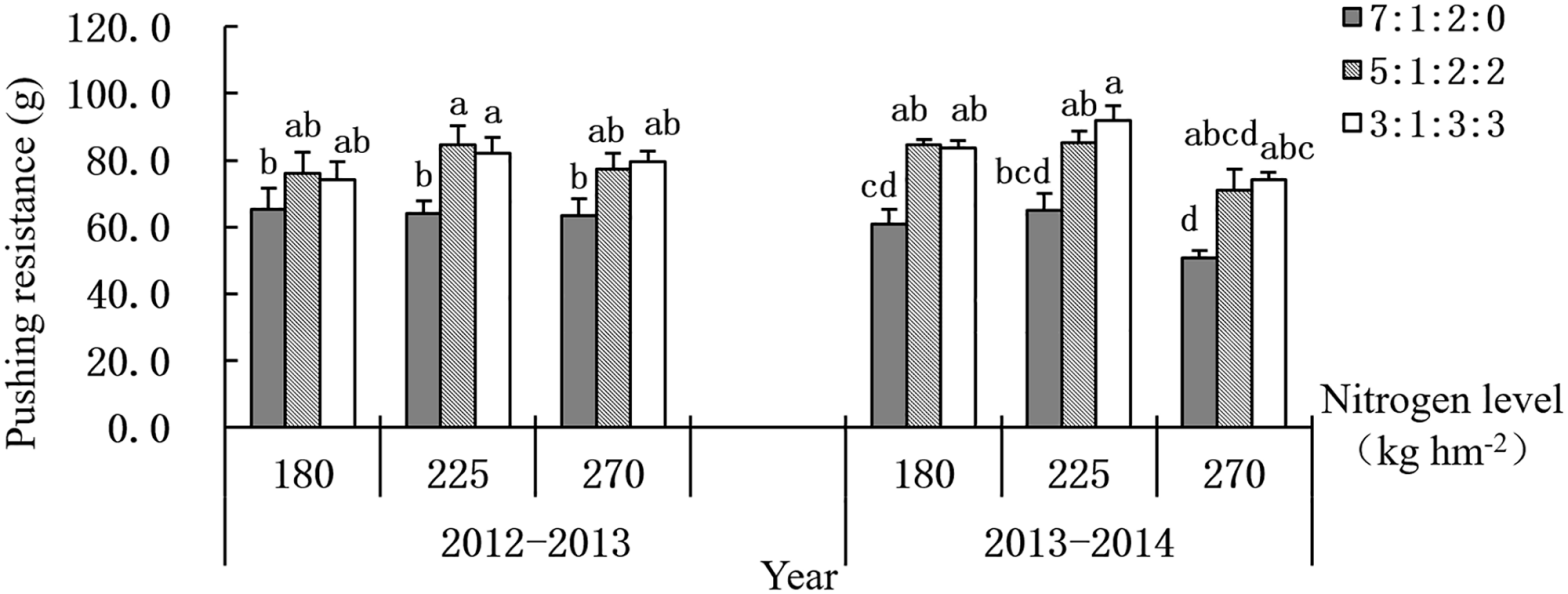
Effect of nitrogen levels and ratios on pushing resistance. Different letters on the culm mean significant difference among the treatments at P < 0.05 as determined by the LSD test. The test was conducted separately between the two years.

It can be seen from Fig 2 that the pushing resistance increased first and then decreased with the increase of nitrogen application rate. The pushing resistance increased with the decrease of basal nitrogen fertilizer ratio under the nitrogen levels were 225 kg ha^-1^ and 270 kg ha^-1^. However, when the nitrogen level was 180 kg ha^-1^, it reached the highest at the treatment of 5:1:2:2, followed by the treatment of 3:1:3:3, and the lowest at the treatment of 7:1:2:0. There was no significant difference between nitrogen ratios of 5:1:2:2 and 3:1:3:3, but both of them were significantly higher than that of 7:1:2:0 treatment.

The correlation analysis showed that there was a significant negative correlation between the pushing resistance of wheat stem and CLRI, and the equation was y = 0.2211x + 10.383(r = -0.68**), indicating that it is feasible of using pushing resistance as an indicator to measure the lodging resistance of wheat.

### 3.4 Plant height and basal internode length

As shown in Table 3, the plant height of Yangmai 20 increased with the augment of nitrogen application doses under the same nitrogen ratio. In addition, with the increase of the proportion of basal fertilizer, the plant height decreased, but N ratio of 5:1:2:2 was higher than that of 3:1:3:3 treatment at the medium fertility condition during 2013–2014. The differences between treatments were at the 0.05 probability levels. Analysis of variance showed that nitrogen level had a significant regulating effect on plant height, while the effect of nitrogen ratio, and the interaction effect between nitrogen level and nitrogen ratio had no significant effect on plant height.

**Table 3:** Effect of nitrogen levels and ratios on plant height and length of basal internodes.

N level (kg ha^-1^)	N ratio	2012–2013				2013–2014			
		Length of basal internodes (cm)		Plant height (cm)	Length of basal internodes/Plant height (%)	Length of basal internodes (cm)		Plant height (cm)	Length of basal internodes/Plant height (%)
		I	II			I	II		
180	7:1:2:0	6.71abc ^α^	10.63ab	90.54a	18.06ab	5.72abc	9.53b	92.34a	18.11ab
	5:1:2:2	6.44bc	10.48ab	90.93a	17.56ab	5.49bc	9.48b	92.79a	17.66ab
	3:1:3:3	5.99c	10.46b	91.27a	17.11b	5.16c	9.45b	93.47a	17.18b
225	7:1:2:0	7.02ab	10.84ab	91.54a	18.32a	5.92ab	10.13ab	93.63a	18.86ab
	5:1:2:2	6.57bc	10.61ab	91.68a	17.69ab	5.61abc	9.78b	94.64a	17.77ab
	3:1:3:3	6.08c	10.48b	92.02a	17.36ab	5.49bc	9.71b	94.25a	17.20b
270	7:1:2:0	7.50a	10.93a	92.09a	18.45a	6.06a	10.71a	95.25a	19.69a
	5:1:2:2	7.33ab	10.65ab	92.06a	17.96ab	5.88ab	9.79b	95.70a	18.45ab
	3:1:3:3	6.70abc	10.48b	93.04a	17.18b	5.51abc	9.92ab	96.30a	17.76ab
F									
Nitrogen levels(NL)		14.44**	2.87	5.30**	1.28	6.94**	8.15**	4.85**	4.40*
Nitrogen ratio(NR)		14.26**	11.11**	1.35	16.39**	14.08**	5.31*	0.55	10.01**
NL×NR		0.19	0.70	0.16	0.30	0.31	2.26	0.03	0.44

^α^ Values in a column followed by the same letter are not significantly different at P < 0.05 as determined by the LSD test. The test was conducted separately between the two years.

* F-test significant at 5% level.

** F-test significant at 1% level.

The effect of nitrogen fertilizer on the basal internodes length of Yangmai 20 was significant (Table 3). Data of the two years showed that the length of the first and second basal internodes showed an increasing trend with the augment of nitrogen level. The average lengths of the first and second basal internodes under the high and medium fertility conditions was 3.33%, 2.18% (225 kg ha^-1^) and 9.78%, 3.72%(270 kg ha^-1^), respectively, longer than that of the low fertility condition (180 kg ha^-1^). Under the same nitrogen level, the variation of the basal internode length was opposite to the plant height with the increase of the topdressing ratio, and the differences between treatments was at the 0.05 probability levels. The ratio between basal internodes length and plant height increased with the augment of nitrogen level and basal fertilizer ratio, except in the nitrogen ratio of 3:1:3:3, where the ratio increased first and then decreased with the augment of nitrogen level. The results of variance analysis showed that the effect of nitrogen level and nitrogen ratio on the length of the first internode was at the 0.01 or 0.05 probability levels, while the nitrogen ratios had significant effect on the length of the second internode.

The above results indicated that excessive application of nitrogen fertilizer and superfluous basal fertilizer can easily cause excessive elongation of the basal internodes and even lodging.

### 3.5 Stem diameter, wall thickness and filling degree of the basal internodes

It can be seen from Table 4 that under the same nitrogen ratio, the diameter of the first basal internode decreased with the increase of nitrogen level, but the difference was not significant. The trend of the first basal internode diameter among different nitrogen ratios was 3:1:3:3>5:1:2:2>7:1:2:0 (the ratio of nitrogen applied before sowing, at tillering, jointing and booting stage), of which, the diameter of 3:1:3:3 treatment increased by 7.68% and 1.98% compared with the treatments of 5:1:2:2 and 7:1:2:0. Analysis of variance showed that nitrogen level affect the diameter of the first basal internode at the 0.01 or 0.05 probability levels, with the F value to be 24.886** (2012–2013) and 7.116* (2013–2014), respectively. The trend of the second internode was the same as that of the first internode, but the treatments had no significant difference.

**Table 4:** Effect of nitrogen levels and ratios on diameter, wall thickness and filling degree of basal internodes.

N level (kg ha ^-1^)	N ratio	2012–2013						2013–2014					
		Diameter (mm)		Wall thickness (mm)		Filling degree (mg DW cm^-2^)		Diameter (mm)		Wall thickness (mm)		Filling degree (mg DW cm^-2^)	
		I	II	I	II	I	II	I	II	I	II	I	II
180	7:1:2:0	4.20ab ^α^	4.56a	0.62ab	0.55ab	23.34ab	22.91abc	4.51ab	4.96a	0.60abc	0.53b	22.10abc	21.20ab
	5:1:2:2	4.49ab	4.86a	0.65ab	0.63a	28.80ab	30.28abc	4.58ab	5.06a	0.63abc	0.55ab	26.07ab	25.75a
	3:1:3:3	4.60a	4.89a	0.66a	0.63a	34.45a	33.69a	4.83a	5.17a	0.69a	0.61a	26.49a	25.37ab
225	7:1:2:0	4.15ab	4.55a	0.58ab	0.53b	22.00b	20.71bc	4.46ab	4.94a	0.58bc	0.55ab	20.58bc	20.86ab
	5:1:2:2	4.57a	4.80a	0.64ab	0.63a	28.13ab	28.52abc	4.57ab	4.96a	0.61abc	0.57ab	24.65ab	24.07ab
	3:1:3:3	4.60a	4.85a	0.66a	0.63a	32.01ab	32.09ab	4.54ab	4.97a	0.64ab	0.58ab	24.05abc	23.38ab
270	7:1:2:0	4.03b	4.48a	0.56b	0.53b	20.40b	20.08c	4.44b	4.86a	0.54c	0.53b	18.69c	19.67b
	5:1:2:2	4.47ab	4.80a	0.61ab	0.61ab	26.85ab	25.66abc	4.56ab	4.92a	0.58bc	0.53b	21.41abc	20.22ab
	3:1:3:3	4.52a	4.77a	0.62ab	0.61ab	29.32ab	28.68abc	4.68ab	4.95a	0.56bc	0.53b	22.13abc	22.55ab
F													
Nitrogen levels(NL)		1.37	0.59	5.33*	0.76	1.82	2.89	2.36	4.10	7.60*	6.53*	12.50**	7.07*
Nitrogen ratio(NR)		24.89**	9.18**	11.10**	29.05**	16.46*	18.22**	7.12*	2.02	16.27*	6.24*	13.08**	7.73*
NL×NR		0.27	0.06	0.61	0.08	0.14	0.13	1.26	0.47	1.46	2.18	0.22	1.10

^α^ Values in a column followed by the same letter are not significantly different at P < 0.05 as determined by the LSD test. The test was conducted separately between the two years.

* F-test significant at 5% level.

** F-test significant at 1% level.

The effect of nitrogen fertilizer on the wall thickness of the stem was as follows: the wall thickness of stem decreased with the increase of nitrogen level generally (Table 4). Under the same condition of nitrogen application, the trend of the wall thickness among different nitrogen ratios was 3:1:3:3>5:1:2:2>7:1:2:0, but treatments of 3:1:3:3 and 5:2:2:2 had no significant difference. The trend of the filling degree was similar to the wall thickness of the first and second basal internodes. Correlation analysis demonstrated that the wall thickness and filling degree of the basal internodes were negatively and significantly correlated with the lodging score, and the correlation coefficient (r) were -0.87**, -0.77** (the wall thickness of first and second basal internodes) and -0.80**, -0.75** (the filling degree of first and second basal internodes) respectively. So, the wall thickness and filling degree of stem were closely related to the lodging. It suggested that reducing the amount of nitrogen fertilizer, especially the amount of nitrogen in the early stage, could enhance the lodging-resistance of wheat by increasing the wall thickness and filling degree of stem.

### 3.6 Water-solute carbohydrates (WSC) and nitrogen (N) content and WSC/N ratio

With the increase of nitrogen level, the nitrogen content of the stem was significantly increased and showed a downtrend with the decrease of basal fertilizer at low nitrogen level (180kg ha^-1^). When the amount of nitrogen application was 225 kg ha^-1^ and 270 kg ha^-1^, the excessive proportion of basal fertilizer should result in high nitrogen content in the stem, and the appropriate postponing of basal fertilizer could decrease the nitrogen content. However, if the ratio of nitrogen at the booting stage (DC 41) was too high, it also could lead to the increase of nitrogen content. Therefore, the nitrogen content of the stem in the treatment of 5: 1: 2: 2 was lower than the other two nitrogen ratio treatments. Variance analysis showed that nitrogen application rate had a significant effect on the nitrogen content of stem, but the effect of nitrogen ratio and the interaction among them was not significant (Table 5).

**Table 5:** Effect of nitrogen levels and ratios on nitrogen content, water-solute carbohydrates and WSC/N^α^ of stem.

N level (kg ha ^-1^)	N ratio	2012–2013			2013–2014		
		Nitrogen content (%)	Water-solute carbohydrates (%)	WSC/N	Nitrogen content (%)	Water-solute carbohydrates (%)	WSC/N
180	7:1:2:0	0.680bc ^β^	15.93ab	23.43ab	0.703cd	15.45a	21.98ab
	5:1:2:2	0.643c	17.08a	26.57a	0.688d	16.87a	24.52a
	3:1:3:3	0.634c	16.62a	26.21a	0.672d	16.32a	24.29a
225	7:1:2:0	0.705abc	14.30abc	20.29bc	0.740abcd	13.87ab	18.74bc
	5:1:2:2	0.678bc	15.08ab	22.25ab	0.712cd	14.63ab	20.55ab
	3:1:3:3	0.688abc	16.03ab	23.30ab	0.722bcd	15.55a	21.52ab
270	7:1:2:0	0.789a	11.35c	14.38d	0.797ab	11.99b	15.04c
	5:1:2:2	0.764ab	13.01bc	17.03cd	0.781abc	13.67ab	17.51bc
	3:1:3:3	0.777ab	14.59abc	18.77bcd	0.809a	14.15ab	17.49bc
F							
Nitrogen levels(NL)		26.61**	21.76**	54.52**	34.53**	15.07**	38.78**
Nitrogen ratio(NR)		1.67	6.15**	8.98**	1.14	4.85*	6.22**
NL×NR		0.19	1.10	0.36	0.66	0.42	0.11

^α^ WSC/N: the ratio of water-solute carbohydrates to nitrogen

^β^ Values in a column followed by the same letter are not significantly different at P < 0.05 as determined by the LSD test. The test was conducted separately between the two years.

* F-test significant at 5% level.

** F-test significant at 1% level.

The laws of water-solute carbohydrates and WSC/N ratio were the same basically (Table 5). Both the water-solute carbohydrates and WSC/N ratio showed downtrend with the increase of nitrogen level, indicating that immoderate nitrogen supply would lead to soaring of free nitrogen in leaves, and photosynthetic products were converted into protein, affecting the accumulation of carbohydrates in the stem. The results of variance analysis showed that the amount of nitrogen and nitrogen ratio had significant or extremely significant effect on the water-solute carbohydrates and WSC/N ratio of the stem at milk stage (DC 73). High WSC/N ratio indicated that the carbohydrates was sufficient in the stem, which was beneficial to lodging resistance of wheat. Therefore, it is possible to reduce the amount of nitrogen fertilizer and use appropriate ratio of sugar to nitrogen to achieve the purpose of improving the lodging resistance of wheat.

### 3.7 Lignin, cellulose and silicon content

**Fig 3:**
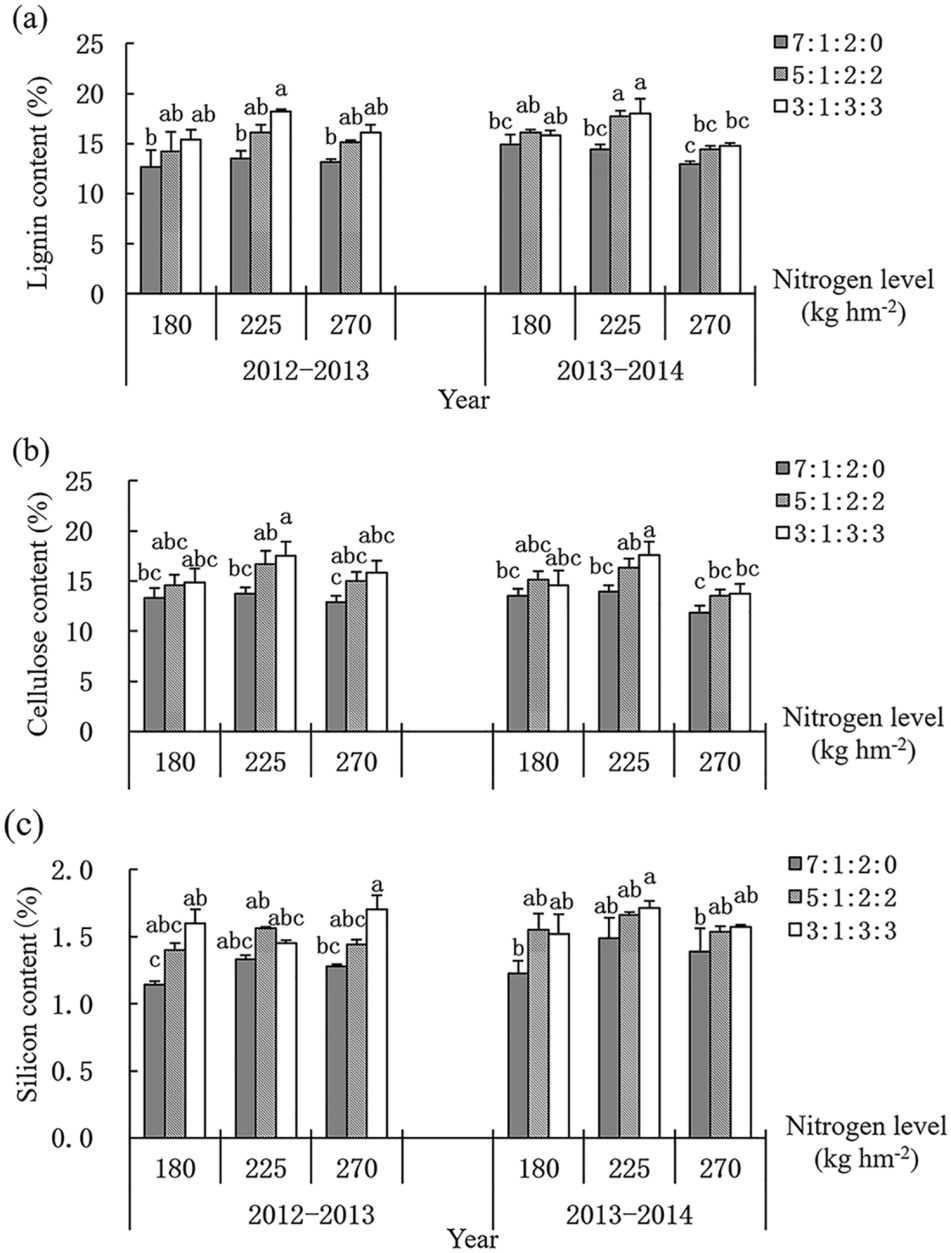
Effect of nitrogen levels and ratios on lignin (a), cellulose (b) and silicon (c). Different letters on the culm mean significant difference among the treatments at P < 0.05 as determined by the LSD test. The test was conducted separately between the two years.

The lignin content increased first and then decreased with the increase of total nitrogen level (Fig 3). Compared with the treatment of 7: 1: 2: 0, postponing basal nitrogen application (the treatments of 5: 1: 2: 2 and 3: 1: 3: 3) could increase the lignin content of the basal internodes significantly under 225 kg ha^-1^ and 270 kg ha^-1^ nitrogen levels, while the difference among different nitrogen treatments had no significant difference under 180 kg ha^-1^ nitrogen level. This indicated that high proportion of basal fertilizer will lead to the reduction of lignin content in basal internodes. The content of cellulose in basal internodes was basically consistent with that of lignin, which showed the trend of increased first and then decreased along with the increase of total nitrogen level. The increased proportion of topdressing fertilizer would also result in the increase of cellulose content (Fig 3).

Silicon mainly exists in the cell wall of epidermal cells and abundant silicon can enhance the hardness, toughness and elasticity of the stem. The content of silicon increased first and then decreased with the increase of total nitrogen application. Reducing the proportion of basal nitrogen (the treatments of 5: 1: 2: 2 and 3: 1: 3: 3) could increase the silicon content of the basal internodes compared with the treatment of 7: 1: 2: 0. The results of variance analysis showed that the effect of nitrogen ratio on the silicon content was significant, while the nitrogen level only significantly affected the silicon content in 2013–2014 (Fig 3).

It was suggested that the moderate increase of nitrogen level was beneficial to improve the content of lignin, cellulose and silicon in basal internodes, but the proportion of basal fertilizer should be reduced, which could enhance the lodging resistance of wheat.

### 3.8 Correlation analysis and grey relational analysis

Correlation analysis demonstrated that stem wall thickness and filling degree of first and second basal internodes, lignin, cellulose and WSC/N ratio positively correlated with culm lodging resistance index (CLRI) significantly. Remarkable negatively correlations were also found between the length of first internode and CLRI. Stem wall thickness were significantly positively associated with filling degree, lignin, cellulose and WSC/N ratio of the basal internodes. Stem wall thickness and filling degree of first and second basal internodes were also significantly (P < 0.05) negatively correlated with plant height. Interestingly, the diameter of basal internodes did not correlate with the length of first basal internode, while, negatively and significantly (P < 0.01) correlated with the length of second basal internode (Table 6).

**Table 6:** Phenotypic correlations for culm lodging resistance index, lodging score and culm characters^α^.

	CLRI	LS	PH	L-I	L-II	D-I	D-II	WT-I	WT-II	F-I	F-II	LC	CC	SC	WSC/N
CLRI	1.00														
LS	-0.79**	1.00													
PH	-0.44	0.54*	1.00												
L-I	-0.67**	0.74**	0.77**	1.00											
L-II	-0.23	0.13	-0.49*	-0.40	1.00										
D-I	0.52*	-0.39	0.41	0.05	-0.65**	1.00									
D-II	0.40	-0.22	0.49*	0.30	-0.83**	0.91**	1.00								
WT-I	0.90**	-0.87**	-0.46*	-0.69**	-0.22	0.51*	0.38	1.00							
WT-II	0.78**	-0.77**	-0.47*	-0.76**	0.18	0.44	0.18	0.81**	1.00						
F-I	0.85**	-0.80**	-0.49*	-0.81**	0.09	0.42	0.17	0.80**	0.90**	1.00					
F-II	0.86**	-0.75**	-0.47*	-0.78**	0.13	0.43	0.17	0.77**	0.90**	0.98**	1.00				
LC	0.59**	-0.54*	0.15	-0.26	-0.46*	0.62**	0.53*	0.58**	0.52*	0.54*	0.51*	1.00			
CC	0.66**	-0.68**	-0.13	-0.52*	-0.20	0.43	0.28	0.66**	0.68**	0.65**	0.62**	0.92**	1.00		
SC	0.34	-0.25	0.48*	0.01	-0.52*	0.70**	0.64**	0.28	0.35	0.35	0.33	0.75**	0.62**	1.00	
WSC/N	0.89**	-0.77**	-0.53*	-0.57*	-0.27	0.37	0.33	0.82**	0.55*	0.67**	0.67**	0.34	0.43	0.10	1.00

^α^ CLRI: culm lodging resistance index; LS: lodging score; PH: plant height; L-I: length of first basal internode; L-II: Length of second basal internode; D-I: diameter of first basal internode; D-II: diameter of second basal internode; WT-I: wall thickness of first basal internode; WT-II: wall thickness of second basal internode; F-I: filling degree of first basal internode; F-II: filling degree of second basal internode; LC: lignin content; CC: cellulose content; SC: silicon content; WSC/N: the ratio of water-solute carbohydrates to nitrogen.

* F-test significant at 5% level.

** F-test significant at 1% level.

In order to study the influence degree of different culm traits on lodging resistance, grey relational analysis for culm lodging resistance index (CLRI) and stem characters were carried out (Table 7). According to the order of the gray relational degree, stem wall thickness of first internode, WSC/N ratio, filling degree of second basal internodes, stem wall thickness of second internode, filling degree of first basal internodes and lignin content were determined the significant factors. The results showed that through nitrogen regulating stem wall thickness, filling degree, water-solute carbohydrates and lignin of the basal internodes could effectively improve the culm lodging resistance index of the cultivar and enhance the lodging resistance.

**Table 7:** Grey relational analysis for culm lodging resistance index and culm characters.

Culm characters	Grey correlation degree (p = 0.5)	Ranking
Plant height	0.345	11
Length of first basal internode	0.299	13
Length of second basal internode	0.343	12
Diameter of first basal internode	0.362	9
Diameter of second basal internode	0.360	10
Wall thickness of first basal internode	0.634	1
Wall thickness of second basal internode	0.550	4
Filling degree of first basal internode	0.550	5
Filling degree of second basal internode	0.562	3
Lignin content	0.485	6
Cellulose content	0.448	7
Silicon content	0.388	8
The ratio of water-solute carbohydrates to nitrogen	0.567	2

## Discussion

Lodging is one of detrimental constraints limiting wheat yields and quality by bending or breaking stems on wheat production worldwide [15]. The cause of the lodging is diverse, including cultivar traits, climate and environment, influence of plant diseases and insect pests, and inappropriate cultivation measures. In the past, lodging resistance has been improved by shortening stems through the use of dwarfing genes (Rht1 and Rht2). But, several studies have shown that yield will reduced when plants are shortened too much [6, 36, 37]. So further height reductions could have little future application as selection criteria. General feeling among researchers working on lodging was that a shorter cultivar will lodge less compared to the tall ones. However, Zuber et al. [16] have reported cultivars with the same height can differ in standing power, and Hobbs et al. [38] even found a semi dwarf variety lodges less than a double dwarf variety. These supported the observation that the quality of wheat culm might be more important for lodging resistance rather than plant height alone. Wheat culms are erect, cylindrical, jointed and consist of five to six internodes. The basal internode is very short, the second internode elongates somewhat more and each successive internode elongates progressively more. The basal part of the culm plays an important role in lodging resistance as it provides a lever to hold the plant upright [8]. Previous studies showed that lodging resistance was significantly correlated with some morphological and chemical characteristics of culm [3, 21], Significant correlations between the lodging score and several morphological and anatomical traits were found for internode length, stem diameter, stem weight per cm, width of mechanical tissue, weight of low internodes, and width of stem walls. Culm chemical components including cellulose and lignin content, the amount of carbohydrate stored in culm, and quantity of silicon and potassium were positively related with lodging resistance [16, 19].

In the present study, culm lodging resistance index (CLRI) is an important parameter to measure the lodging resistant ability of crops and to evaluate the lodging risk in the agricultural production. Correlation analysis demonstrated that stem wall thickness and filling degree of basal internodes, lignin, cellulose and WSC/N ratio were positively and significantly correlated with CLRI. Remarkable negatively correlations were also found between the length of first internode and CLRI. While, plant height, the length and diameter of second internode and silicon of basal internode had no significantly correlation with CLRI, which is different from several previous studies [3, 16, 19]. The result maybe duo to the field has high levels of soil mineral nitrogen. Therefore increasing nitrogen rate and basal fertilizer have less influence to plant height, the length and diameter of second internode and silicon of basal internode than other characteristics of culm.

The application of nitrogen could effected lodging risk and grain yield significantly. The increase in the nitrogen rates promoted higher lodging, affected the grain and flour quality and had no effect on yield [26]. In Egypt, lodging reduced the grain yield by 19.9 and 7.2% at 225 and 275 kg N ha^-1^ as compared to 150 and 175 kg N ha^-1^ application, respectively [25, 27]. The use of high N rates increased lodging with resultant yield losses between 7 and 35% in Mexico and 12–66% in India [16]. Fischer and Stapper [4] reported that controlling the N fertility under a certain limit could improve the lodging resistance and ultimately reduce the likelihood of lodging. Berry et al. [7] suggested that delay nitrogen fertilizer applications decrease the likelihood of lodging. Similar findings were reported by Peake et al. [28].

Recent work has shown that the morphological and anatomical traits of culm with beneficial effects on lodging may be changed by reducing and delaying applications of fertilizer, such as wide stem bases with thick walls, high lignin and cellulose content and efficient canopy structure which can increase light interception at the base of plant [7, 39]. Increasing the nitrogen fertility beyond the threshold will promote vegetative growth, increase leaf area index, biomass and tiller count and reduces light interception at the base of plant [3, 27]. The cumulative effect of all these factors produces lanky and succulent culms that are highly susceptible to lodging stress and ultimately decreases grain yield and its components. Crook and Ennos [23] reported that high levels of nitrogen increased the height of the stem, thereby increasing the 'self-weight' moment transmitted into the ground and weakened the stems. Kheiralla et al. [27] found that application of N beyond 175 kg ha^-1^ decreased the stem diameter, dry weight per unit length and stem wall thickness.

The present field experiment was conducted to study the effects of different nitrogen management on lodging resistance and yield potential and to determine whether nitrogen management can reduce lodging risk without reducing yield potential finally. The result shown that grain yield response to nitrogen doses was quadratic and grain yield reached the highest at 225 kg ha^-1^ nitrogen level. The treatment of 5:1:2:2 increased by 10.05% and 2.27% compared with the treatments of 7:1:2:0 and 3:1:3:3, respectively. As the increase of nitrogen level and basal nitrogen ratio, the basal internodes became slender and fragile, and the stem wall thickness, filling degree and the strength of the stem were decreased gradually, which increased the lodging risk significantly. The results were similar to previous studies. In a word, choosing the moderate nitrogen level and reducing the basal fertilizer ratio could increase the stem strength and toughness and the culm lodging resistance effectively and got the higher grain yield simultaneously.

In conclusion, for Yangmai 20, the planting density of 180×10^4^ ha^-1^, nitrogen level of 225 kg ha^-1^, and the proportion of 5: 1: 2: 2 (the ratio of nitrogen amount applied before sowing, at tillering, jointing and booting stage) effectively reduced plant height and the length of basal internodes, increased the wall thickness, filling degree, the WSC/N ratio and the contents of lignin and cellulose in basal internodes. Moreover, this combination of planting density and nitrogen level and ratio also increased the lodging resistance and grain yield.

## Supporting information

S1 Table Meteorological data of experimental location. (XLSX)

S2 Table The soil nutrient content of the test site. (XLSX)

S3 Table Date of height of center of gravity: Used for calculating culm lodging resistance index. (XLSX)

S4 Table Date used for correlation analysis. (XLSX)
